# Targeting Src tyrosine kinase to enhance radioiodide uptake in breast cancer

**DOI:** 10.1530/ERC-24-0312

**Published:** 2025-08-20

**Authors:** Vikki L Poole, Mohammed M Alshahrani, Selvambigai Manivannan, Iñigo Landa, Aditi Hariharan, Rebecca J Thompson, Merve Kocbiyik, Caitlin E M Thornton, Katie Brookes, Alice Fletcher, Kristien Boelaert, Martin L Read, Christopher J McCabe, Vicki E Smith

**Affiliations:** ^1^ Department of Metabolism & Systems Science (MSS) and Centre for Endocrinology, Diabetes and Metabolism (CEDAM), College of Medicine and Health, University of Birmingham, Birmingham, UK; ^2^ Institut Gustave Roussy, Inserm U981, Villejuif, France; ^3^ IHU-National PRecISion Medicine Center in Oncology, Villejuif, France; ^4^ Department of Applied Health Sciences, College of Medicine and Health, University of Birmingham, Birmingham, UK

**Keywords:** NIS, radioiodide, PBF/PTTG1IP, Src myristoylation, breast cancer

## Abstract

Sodium iodide symporter (NIS) expression in breast cancer renders radioiodide (RAI) a promising treatment modality. However, insufficient functional NIS within the plasma membrane limits RAI uptake (RAIU). We aimed to elucidate NIS regulatory mechanisms that impede RAIU in breast cancer and identify molecular targets for stimulating RAI-avidity in breast tumours. Mechanistic interaction between pituitary tumor-transforming gene-binding factor (PBF/PTTG1IP) and NIS was investigated through NanoBiT, co-immunoprecipitation, immunofluorescent microscopy, subcellular localisation and RAIU assays utilising wild-type and CRISPR-Cas9 PBF knockout breast cancer cells. In breast cancer cells, NIS:PBF interaction resulted in diminished RAIU, reversible through reduced PBF phosphorylation by the Src inhibitor dasatinib. Src overexpression diminished RAIU in a PBF-dependent manner that was mediated by Src myristoylation by N-myristoyltransferase 1 (NMT1). NMT1 inhibition significantly enhanced RAIU via Src and PBF in breast and thyroid cancer cells. Bioinformatic analyses revealed clinical associations between high Src and NMT1 expression and increased tumour recurrence in RAI-treated thyroid cancers indicating RAI-resistance. In breast cancer, high PBF and Src expression was associated with the more aggressive tumours that are most likely to benefit from targeted RAI therapy. We describe a new NIS regulatory pathway in breast cancer cells via Src myristoylation and PBF phosphorylation and show that the same pathway exists in thyroid cells, the canonical setting for the exploitation of NIS function. These findings reveal that PBF interaction with NIS may be modulated by Src, which in turn is susceptible to NMT inhibition, and suggest that targeting NMT1 may represent an innovative approach for augmenting RAI-avidity in breast cancer.

## Introduction

The sodium iodide symporter (NIS) mediates iodide uptake for thyroid hormone biosynthesis and has been successfully utilised for decades to deliver radioiodide (RAI) as a targeted therapeutic for differentiated thyroid cancer (DTC) (1). DTC patients with RAI-resistant metastatic tumours due to reduced NIS expression have an extremely poor prognosis, as do those with more aggressive forms that are inherently RAI-resistant (RAIR), such as anaplastic thyroid cancer (ATC). New therapeutic strategies seek to re-establish NIS expression to restore RAI uptake (RAIU) (1). Furthermore, tumour-targeted NIS gene therapy is an exciting prospect for non-thyroidal tumours (2).

Breast cancer represents a particularly compelling application for radioiodide treatment due to increased endogenous NIS expression in most tumours (70–80%) (3, 4, 5), including triple negative breast cancer (TNBC) (6) and metastases (7, 8), which are often resistant to standard treatments. However, few NIS-positive tumours accumulate RAI (6, 7, 8, 9). This disparity between NIS expression and function suggests inadequate NIS plasma membrane (PM) localisation (10). Thus, understanding how NIS is trafficked in breast cancer cells is crucial to facilitate RAIU.

Diminished NIS PM targeting/retention also contributes to RAIR thyroid cancer and limits strategies to reinduce NIS expression (11). Despite the essential role of membranous NIS in mediating thyroid hormone production and thyroid cancer treatment, its regulation remains to be fully elucidated (11). Our recent work identified endocytosis as a critical determinant of NIS function and, ultimately, patient outcome in thyroid cancer (12).

We identified the first NIS-interacting protein that modulates NIS internalisation and reduces RAIU in thyroid cancer (13). Pituitary tumor-transforming gene-binding factor (PBF/PTTG1IP) is upregulated in thyroid cancer and associated with poorer disease outcomes (14, 15). PBF post-translationally regulates NIS function through its binding and endocytosis (13). Subsequently, PBF tyrosine residue 174 (Y174) was found to mediate this effect. We showed that the tyrosine kinase Src could induce PBF phosphorylation at Y174 (PBF-pY174) and, importantly, Src inhibition overcame PBF repression of NIS and restored RAIU in thyroid cancer cells (16). More recently, Src has also been identified as a NIS-interacting protein (17, 18).

Here, we investigated whether PBF impedes RAIU in breast cancer cells, given its concurrent upregulation in breast tumours (19), and elucidated the underlying mechanisms of repression. We show that phosphorylated PBF represses RAIU while the Src inhibitor dasatinib restores NIS function in breast cancer cells by inhibiting PBF-pY174. Furthermore, we demonstrate that Src myristoylation, a lipid modification that promotes PM association, mediates RAIU repression via PBF in both breast and thyroid cancer cells, and RAIU is significantly induced by N-myristoyltransferase 1 (NMT1) inhibition. This study reveals that myristolyated Src can repress RAIU via PBF and highlights NMT1 as a potential therapeutic target for the induction of RAIU in breast tumours and the restoration of NIS function in thyroid cancer.

## Materials and methods

### Cell lines

Breast cancer (MCF-7 and MDA-MB-231) and thyroid cancer (TPC-1) cell lines were maintained in RPMI-1640 medium (Thermo Fisher Scientific, USA). TPC-1 cells were obtained from the CU Cancer Center Tissue Culture Shared Resource (University of Colorado). All other cell lines were acquired from ECACC (European Collection of Authenticated Cell Cultures). Cells were supplemented with 10% fetal bovine serum (Thermo Fisher Scientific), penicillin (10^5^ U/L), and streptomycin (100 mg/L) and maintained at 37°C and 5% CO_2_. A TPC-1 cell line stably expressing NIS has been described previously and was maintained in RPMI supplemented with blasticidin S (15 μg/mL) (20). Cells were used at low passage (<35) and authenticated by short tandem repeat analysis (Northgene, UK).

### Plasmids and transfection

Plasmids containing the full-length cDNA for haemagglutinin (HA)-tagged PBF, PBF-Y174A mutant, MYC-tagged NIS and Src have been described previously (13, 14, 16). The PBF-EEN mutant (NP_004330.1: p.Glu170_Asn172delinsAlaAlaAla) was generated using a QuikChange Site-Directed Mutagenesis Kit (Agilent, USA) with the primers 5′-GAAAAAAATATGGCCTGTTTAAAGCAGCAGCCCCGTATGCTAGATTTG-3′ and 5′-CAAATCTAGCATACGGGGCTGCTGCTTTAAACAGGCCATATTTTTTTC-3′. Nucleotide substitutions are underlined. The Src-T341I mutant was similarly generated using the primers 5′-GAGCCCATTTACATCGTCATCGAGTACATGAGCAAGGGG-3′ and 5′-CCCCTTGCTCATGTACTCGATGACGATGTAAATGGGCTC-3′. For the NanoBiT assay, NIS and PBF cDNA were each tagged at the 3′ end with sequences encoding either SmBiT or LgBiT (Promega, USA) within pcDNA3.1+ (Invitrogen, USA) (12).

Plasmid and siRNA transfections were performed with TransIT^®^-LT1 reagent (Mirus Bio, USA) and RNAiMAX (Thermo Fisher), respectively, following manufacturer’s protocols. For stable expression, cells transfected with wild-type (WT) and mutated PBF were selected and maintained in 1 mg/mL G418. For stable PBF knockdown, cells were transduced with lentiviral particles containing shRNA targeting PBF mRNA (SMARTvector Human Lentiviral PTTG1IP – VSH6063/SH-011820; Dharmacon, USA) alongside control shRNA (SMARTvector Non-targeting hCMV-TurboGFP Control Particles – S-005000-01; Dharmacon). For Src knockdown, the ON-TARGETplus Human SRC siRNA SMARTPool was used at a final concentration of 100 nM (Dharmacon).

### Antibodies and reagents

The following antibodies were used: mouse monoclonal anti-HA.11 (16B12) (Biolegend, USA), rabbit monoclonal anti-HA (C29F4) (Cell Signaling Technology, USA), rabbit PBF-pY174 (CovalAb, France) (16), mouse monoclonal anti-Myc-Tag (9B11) (Cell Signaling Technology), rabbit polyclonal anti-NIS (24324-1-AP) (Proteintech, USA), rabbit monoclonal anti-Src (32G6; 2123) (Cell Signaling), rabbit polyclonal anti-Src-pY418 (ab4816) (Abcam, UK), rabbit polyclonal anti-PBF (Eurogentec, Belgium) (16), rabbit polyclonal anti-PBF (T1205) (Sigma Aldrich, USA) and mouse monoclonal anti–β-actin (clone AC-15) (Sigma).

MCF-7 cells were treated with 100 nM all-trans retinoic acid (ATRA; Sigma) and 1 μM dexamethasone (Sigma) to induce endogenous NIS. Src family kinase inhibitors PP1 (Tocris, UK), dasatinib and saracatinib (Selleck Chemicals, USA) and NMT inhibitor (DDD85646; Drug Discovery Unit, University of Dundee) were dissolved in DMSO to a 10 mM stock concentration. To detect tyrosine phosphorylation, cells were treated with pervanadate (100 μM) for 15 min before protein extraction or immunofluorescence (16).

### NanoBiT live cell protein interaction assays

MCF-7 cells were seeded in 6-well plates and transfected with 1 μg plasmid DNA (NIS-SmBiT + PBF-LgBiT, NIS-LgBiT + PBF-SmBiT and negative control NIS-SmBiT + LgBiT-PRKAR2A (NanoBiT control vector; Promega)). 24 h post-transfection, cells were reseeded into 96-well plates in phenol red-free DMEM (Thermo Fisher). Nano-Glo^®^ Live Cell Reagent (Promega) was added to each well in accordance with the manufacturer’s protocol and measurements were taken every 2 min for 30 min (PHERAstar FS microplate reader; BMG Labtech, Germany).

### Co-immunoprecipitation (co-IP) assays, Western blotting and qPCR

MCF-7 cells in T75 flasks were transiently transfected with either vector only (VO), NIS-MYC + VO or NIS-MYC + PBF-HA. Co-immunoprecipitation assays were performed as described previously (13) with minor modifications. Cells were lysed in 1 mL RIPA buffer with Dounce homogenisation. Each lysate was incubated with 5 μL anti-Myc-Tag antibody and 100 μL Protein-G-Sepharose 4 Fast Flow beads (GE Healthcare Life Sciences, USA) used to capture antibody–protein complexes.

Western blotting was performed as described previously (21). Proteins (30 μg or co-IP eluate) were separated by SDS-PAGE using 15% acrylamide gels (10% for NIS detection). Membranes were probed with anti-Myc-Tag, anti-HA (16B12), anti-PBF-pY174, anti-PBF, anti-Src, anti-Src-pY418 and anti-β-actin antibodies.

Quantitative PCR (qPCR) was performed as described previously using 18s as the internal housekeeping gene for NIS mRNA expression (22) and using the PBF TaqMan Gene Expression Assay (Hs01036322_m1; Thermo Fisher Scientific).

### Immunofluorescence staining

MCF-7 cells were seeded onto coverslips in 6-well plates and transfected with 2 μg DNA. Twenty-four hours post-transfection, cells were washed with PBS, fixed in 4% paraformaldehyde/PBS for 15 min and permeabilised with 0.1% saponin. Cells were incubated for 1 h with primary antibodies (mouse-anti-HA, rabbit-anti-HA, and anti-Myc-Tag) and then secondary antibodies (Alexa-Fluor-555-conjugated goat anti-rabbit and Alexa-Fluor-488-conjugated goat anti-mouse; Invitrogen). Finally, coverslips were mounted onto slides using Prolong Gold anti-fade reagent with DAPI (Molecular Probes) and images were captured on an LSM880 Airyscan confocal microscope (100× objective).

### Radioiodide uptake (RAIU) assays

Cells were seeded into 24-well plates and transfected with 0.5 μg DNA and/or treated with various drugs as stated. RAIU assays were performed 48 h post-transfection, as described previously (16). Before the addition of radioiodide (^125^I), negative control wells were pre-treated for 1 h with the NIS inhibitor, sodium perchlorate (100 μM). All cells were then incubated with 10^−6^ M NaI containing 0.05 μCi ^125^I (specific activity 17.4 mCi/μg; Hartmann Analytic, Germany) for an hour at 37°C and subsequently washed with Hank’s balanced salt solution (HBSS) to remove unincorporated ^125^I. Cells were lysed in 2% SDS and radioactivity was measured in counts per minute using a LKB 1260 Multigamma Gamma Counter. Protein concentration was determined using the Pierce^TM^ bicinchoninic acid (BCA) colourimetric assay (Thermo Fisher Scientific) and results given as picomoles iodide per microgram protein.

### Generation of CRISPR-Cas9 PBF knockout cells

Two 20 bp CRISPR single guide RNA (sgRNA) sequences were designed to target PBF exon 1 via endogenous PAM sequences (Supplementary Fig. 1 (see the section on Supplementary materials given at the end of the article)). Each sgRNA was cloned into the pLentiCRISPR vector BsmBI restriction sites (Addgene #49535 (23)). Parental MCF-7, MDA-MB-231 and TPC-1 cells were transfected with 400 ng pLentiCRISPR-sgRNA#1–2 using TransIT®-LT1 reagent (Mirus Bio), followed by 1 μg/mL puromycin (Sigma) selection for 3 days. Single cells were plated into 96-well plates and the resultant clones were screened by DNA extraction, PCR, T7 Endonuclease I (T7E; New England Biolabs) mismatch assay and Sanger sequencing. For each cell line, two clones were selected with CRISPR-mediated insertion and/or deletion via two different sgRNA sequences, resulting in a frameshift mutation and significant truncation of PBF.

### Gene expression data analyses

Gene expression and clinical data for 59 normal thyroid, 488 papillary thyroid cancer (PTC; THCA), 112 normal breast and 1,197 breast cancer (BRCA) samples from The Cancer Genome Atlas (TCGA) were downloaded from FireBrowse (http://firebrowse.org/) and cBioPortal (https://www.cbioportal.org/) (24, 25). FPKM (fragments per kilobase transcript per million mapped reads) values were transformed as *X* = log_2_ (X + 1) before analysis. Differential gene expression analysis was also performed using the gene expression omnibus (GEO) dataset GSE60542 (26, 27). Expression data were determined by MSK-IMPACT targeted sequencing from 17 poorly-differentiated thyroid cancer (PDTC) and 20 ATC fresh-frozen samples selected from the Memorial Sloan Kettering Cancer Center Pathology Department (1986–2015) according to the classification outlined in (28).

### Patient survival characteristics

Receiver operating characteristic (ROC) curves were plotted in IBM SPSS Statistics (version 29) with the area under the curve (AUC) representing the accuracy of potential gene biomarkers for distinguishing recurrent from non-recurrent disease. Using the classifier evaluation metrics output data, patients were stratified into high and low expression groups for gene biomarkers based on optimal cut-off values for sensitivity and specificity. Disease-free survival (DFS) characteristics (i.e. Kaplan–Meier (log-rank test) and univariate Cox regression analyses) were also determined using optimal expression cut-off values.

### Statistical analyses

Data were analysed using the Student’s *t*-test and Mann–Whitney U test for comparison between two groups of parametric and nonparametric data, respectively. One-way ANOVA with Tukey’s post-hoc test and a Kruskal–Wallis test with Dunn’s post-hoc test were used for comparisons of multiple groups of parametric and nonparametric data, respectively. *P*-values were adjusted using the Benjamini–Hochberg FDR correction procedure to correct for multiple comparisons. Significance was taken as *P* < 0.05. All *P*-values reported from statistical tests were two-sided.

## Results

### Phosphorylated PBF represses RAIU in breast cancer cells

**Figure 1:**
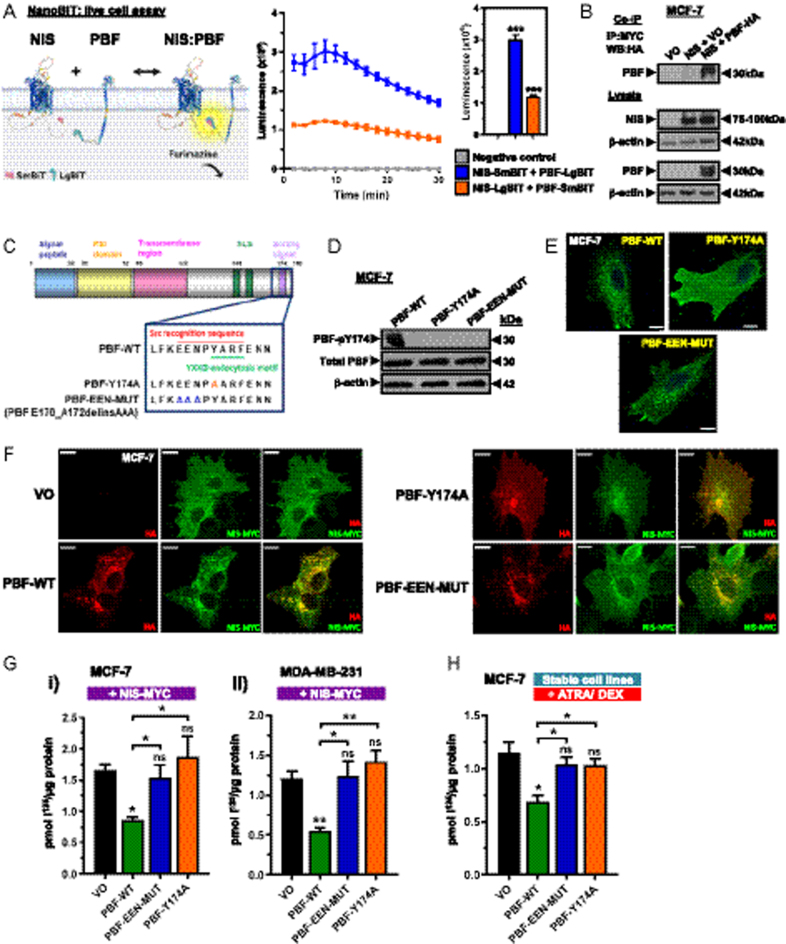
Phosphorylated PBF represses RAIU in breast cancer cells. (A) *Left* – schematic of the interaction between NIS and PBF proteins tagged at the C-terminus with the NanoLuc luciferase subunits LgBiT and SmBiT, respectively. In close proximity, LgBiT and SmBiT form a functional enzyme that uses the substrate furimazine to produce a high-intensity, luminescent signal. Created using Alphafold (59, 60) and BioRender. *Right* – NanoBiT analysis of NIS:PBF interaction in MCF-7 cells, with bar graph showing results at 20 min post-addition of Nano-Glo live cell assay substrate. (B) Co-immunoprecipitation assay showing PBF-HA and NIS-MYC interaction in MCF-7 breast cancer cells. (C) Schematic of PBF highlighting the overlapping phosphorylation site and endocytosis motif at the C-terminus, and the PBF-Y174A and E170_N172delinsAAA (PBF-EEN MUT) mutants. (D) Western blot showing the phosphorylation status of PBF-Y174A and PBF-EEN MUT compared with PBF-WT in MCF-7 cells. (E) Immunofluorescent images showing PBF-WT, PBF-Y174A and PBF-EEN MUT localisation in MCF-7 cells. (F) Immunofluorescent images showing subcellular localisation following the co-transfection of NIS-MYC (green) with VO, PBF-WT, PBF-Y174A or PBF-EEN MUT (red) in MCF-7 cells. Co-localisation is seen in yellow. (G) The effect of PBF and phosphomutants (Y174A/EEN-MUT) on RAIU in MCF-7 (i) and MDA-MB-231 (ii) cells transiently co-transfected with NIS-MYC. (H) The effect of PBF and phosphomutants (Y174A/EEN-MUT) on RAIU in MCF-7 cells treated with ATRA and dexamethasone. VO = vector only control. Bars = 10 μm. *n* = 3 for all experiments. Error bars = SEM. Significance shown compared with VO unless otherwise shown. ns = not significant (*P* > 0.05), * = *P* < 0.05, ** = *P* < 0.01, *** = *P* < 0.001.

Previously, we showed that PBF interacts with NIS in thyroid cancer cells, via PBF-Y174, resulting in diminished RAIU (13, 16). Using NanoBiT assays, we now confirm a robust and dynamic interaction between NIS and PBF in live MCF-7 breast cancer cells (Fig. 1A), supported by co-immunoprecipitation assays (Fig. 1B).

Intriguingly, in addition to a Src-mediated phosphorylation site, PBF-Y174 is the key residue in a tyrosine-based sorting signal (YXXφ; Fig. 1C) required for PBF endocytosis from the PM (16). To specifically assess the impact of PBF phosphorylation, part of the Src consensus sequence preceding Y174 was mutated (PBF E170_N172delinsAAA; PBF-EEN-MUT). Similar to PBF-Y174A, PBF-EEN-MUT lacked Y174 phosphorylation (Fig. 1D), but immunofluorescence microscopy confirmed that, whereas PBF-Y174A showed increased PM accumulation, PBF-EEN-MUT retained subcellular distribution comparable with WT PBF (Fig. 1E). As in thyroid cancer cells, there was extensive co-localisation between NIS and PBF-WT within intracellular vesicles in breast cancer cells, while NIS co-localisation with PBF-Y174A was limited to the PM (Fig. 1F). In contrast PBF-EEN-MUT appeared to co-localise more weakly with NIS, which remained largely expressed at the PM (Fig. 1F).

Importantly, and in line with thyroid cancer cell data, PBF overexpression significantly repressed RAIU both in oestrogen-responsive MCF-7 breast cancer cells and MDA-MB-231 TNBC cells expressing exogenous NIS (Fig. 1G). In contrast, neither PBF-EEN-MUT nor PBF-Y174A altered RAIU compared with VO control (Fig. 1G). All-trans retinoic acid (ATRA) and dexamethasone (Dex) in combination induces NIS expression and RAIU in MCF-7 cells (29) (Supplementary Fig. 2A) and our findings were examined in this model of endogenous NIS expression. ATRA/Dex treatment of MCF-7 cells stably expressing PBF-WT resulted in significantly lower RAIU compared with VO control cells, whereas cells stably expressing PBF-EEN-MUT or PBF-Y174A were comparable with VO cells (Fig. 1H and Supplementary Fig. 2B). Conversely, although a slight increase in uptake was seen, PBF depletion by shRNA knockdown in MCF-7 cells did not significantly alter ATRA/Dex-stimulated RAIU (Supplementary Fig. 2C).

Taken together, these data demonstrate that NIS and PBF interact within breast cancer cells. A similar mechanism of NIS repression to that seen in thyroid cancer occurs in breast cancer cells, whereby PBF binds and internalises NIS, significantly impairing RAIU. Furthermore, additional phospho-mutants confirmed that this is mediated by PBF phosphorylation at Y174.

### Dasatinib potently inhibits PBF phosphorylation and restores PBF-repressed RAIU

**Figure 2:**
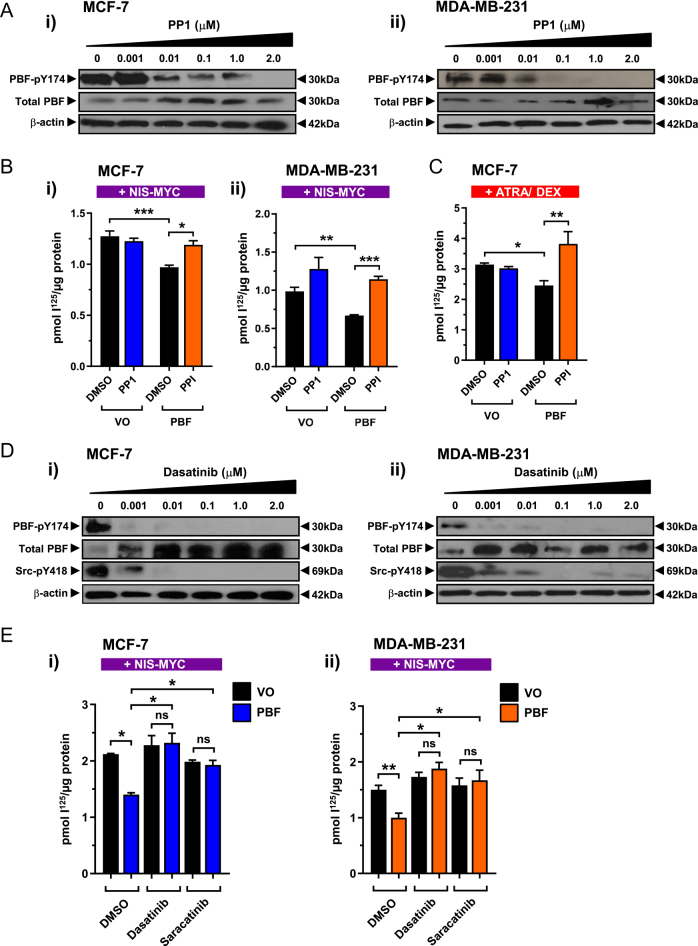
Src inhibition inhibits PBF phosphorylation and restores RAIU. (A) MCF-7 (i) and MDA-MB-231 (ii) cells were treated with varying doses of PP1 (0–2 μM) for 24 h before PBF-pY174 and total PBF expression levels were determined by Western blot. (B) Effect of 2 μM PP1 treatment on PBF-mediated RAIU repression in MCF-7 (i) and MDA-MB-231 (ii) cells transiently co-transfected with NIS-MYC. (C) Effect of 2 μM PP1 treatment on PBF-mediated RAIU repression in MCF-7 cells treated with ATRA and dexamethasone. (D) PBF-pY174 and total PBF expression levels following dasatinib dose response (0–2 μM) treatment for 24 h in MCF-7 (i) and MDA-MB-231 (ii) cells. (E) Effect of 1 nM dasatinib or 10 nM saracatinib treatment on PBF-repressed RAIU in MCF-7 (i) and MDA-MB-231 (ii) cells transiently co-transfected with NIS-MYC. *n* = 3 for all experiments. Error bars = SEM. * = *P* < 0.05, ** = *P* < 0.01, *** = *P* < 0.001.

The Src family kinase (SFK) inhibitor PP1 decreases PBF phosphorylation and restores PBF-mediated RAIU repression in thyroid cancer cells (16). To determine whether SFK inhibition can similarly enhance RAIU in breast cancer cells, MCF-7 and MDA-MB-231 cells were treated with a range of PP1 doses. A marked reduction in PBF phosphorylation was observed from 0.01 μM PP1 treatment in both cell lines (Fig. 2A) and subsequent PP1 treatment restored PBF-mediated RAIU repression in cells expressing exogenous NIS-MYC (Fig. 2B), and ATRA/Dex-induced endogenous NIS (Fig. 2C).

Having confirmed the effectiveness of SKF inhibition at restoring PBF-repressed RAIU in breast cancer cells, we investigated alternative SFK inhibitors to identify a compound that most potently inhibits PBF phosphorylation and therefore has the potential to maximise RAI therapy in both breast and thyroid cancer patients. We selected two SFK inhibitors which target Src with an IC50 <5 nM and are either used clinically or are progressing through clinical trials. MCF-7 and MDA-MB-231 cells were treated with dasatinib and saracatinib at concentrations ranging from 1 nM to 2 μM for 24 h. Dasatinib virtually abolished PBF-pY174 levels at doses starting at 1 nM in both cell lines (Fig. 2D), with saracatinib showing consistent results, albeit at slightly higher doses (Supplementary Fig. 3). Both dasatinib and saracatinib restored RAIU in cells transfected with NIS-MYC and PBF to levels comparable with NIS-MYC and VO co-expression in MCF-7 and MDA-MB-231 cells (Fig. 2E).

Thus, SFK inhibition can overcome PBF repression of RAIU in breast cancer cells and dasatinib was identified as a potent inhibitor of PBF phosphorylation.

### Dasatinib targets Src to restore PBF-mediated RAIU repression

**Figure 3:**
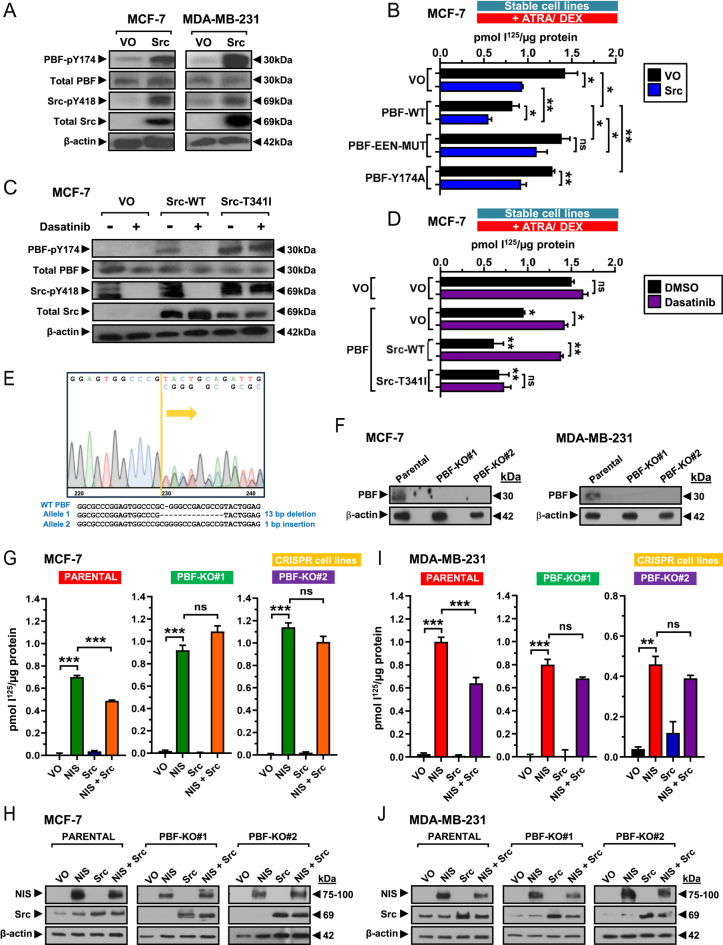
(A) Detection of PBF and PBF-pY174 levels following Src overexpression for 48 h in MCF-7 and MDA-MB-231 cells. (B) RAIU in ATRA/Dex-treated MCF-7 cells with stable expression of VO, PBF-WT, PBF-EEN-MUT or PBF-Y174A following transient transfection with Src or VO control. (C) Expression levels of PBF and PBF-pY174 in MCF-7 cells transiently transfected for 48 h with VO, Src-WT or the gatekeeper mutant Src-T341I and treated for 24 h with either DMSO or 10 nM dasatinib. (D) RAIU in ATRA/Dex-treated MCF-7 cells stably transfected with VO and PBF, transiently transfected with VO, Src-WT or Src-T341I for 48 h and treated with either DMSO or 10 nM dasatinib for 24 h. (E) Example of a heterozygous mutation induced by CRISPR-Cas9 targeting to PBF exon 1 in the MCF-7 PBF-KO #1 clonal cell line. (F) Confirmation of PBF deletion by Western blotting in MCF-7 and MDA-MB-231 PBF-KO cells generated using gRNA#1 (PBF-KO #1) and gRNA#2 (PBF-KO #2). (G) RAIU in parental MCF-7 cells and two PBF knockout cell lines (PBF-KO #1 and #2) following NIS, Src and NIS + Src overexpression. Confirmation of successful transfection shown below by Western blotting. (H and I) RAIU in parental MDA-MB-231 cells and PBF-KO cell lines (PBF-KO #1 and #2) following NIS, Src and NIS + Src overexpression. Confirmation of successful transfection shown below by Western blotting (J). *n* = 3 for all experiments. Error bars = SEM. Significance shown compared with VO unless otherwise shown. ns = not significant (*P* > 0.05), * = *P* < 0.05, ** = *P* < 0.01, *** = *P* < 0.001.

Although a potent SFK inhibitor, dasatinib can also target multiple other kinases (30). To further investigate a role for Src in modulating RAIU in breast cancer cells, we first overexpressed Src in MCF-7 and MDA-MB-231 cells and observed a marked increase in PBF-pY174 expression (Fig. 3A). ATRA/Dex-treated MCF-7 cells stably transfected with VO, PBF-WT, PBF-EEN- MUT or PBF-Y174A were then transiently transfected with Src or VO control. Stable PBF-WT expression significantly reduced RAIU compared with VO-transfected cells, while neither PBF-EEN-MUT nor PBF-Y174A had any marked effect (Fig. 3B). Interestingly, exogenous Src significantly reduced RAIU across each of the stable cell lines compared with VO-transfected cells with the exception of PBF-EEN-MUT (Fig. 3B). Moreover, PBF and Src co-transfection resulted in an additive effect with a significant decrease in RAIU compared with PBF (↓33% compared with PBF + VO, *P* = 0.045) or Src (↓42% compared with VO + Src, *P* = 0.006) alone (Fig. 3B).

To specifically evaluate Src in SFK inhibitor restoration of RAIU, we utilised the Src-T341I gatekeeper mutant, which is resistant to dasatinib. MCF-7 cells were transiently transfected with VO, Src-WT or Src-T341I and treated with either DMSO or 10 nM dasatinib. Dasatinib treatment abrogated levels of active Src-pY418 in Src-WT-expressing cells, whereas cells expressing mutant Src-T341I maintained Src-pY418 levels, confirming drug resistance (Fig. 3C). The induction of PBF-pY174 levels by exogenous Src-WT was abrogated in the presence of dasatinib (Fig. 3C). In contrast, while mutant Src-T341I also markedly increased levels of PBF-pY174, this induction was unaltered by dasatinib (Fig. 3C).

To assess endogenous RAIU, ATRA/Dex-treated MCF-7 cells stably transfected with VO and PBF were transiently transfected with VO, Src-WT or Src-T341I and treated with either DMSO or 10 nM dasatinib (Fig. 3D). Notably, in the presence of Src-T341I, dasatinib was unable to overcome Src’s influence on RAIU (Fig. 3D).

These data strongly suggest that Src is the kinase responsible for PBF phosphorylation and subsequent repression of NIS function, and that dasatinib specifically targets Src to restore PBF-pY174-repressed RAIU.

### Src overexpression represses RAIU in breast and thyroid cancer cells via PBF

Src overexpression significantly repressed RAIU and enhanced PBF repression in breast cells (Fig. 3B). To determine whether Src can modulate NIS function independently of PBF, we generated breast cancer cell lines with CRISPR-Cas9-mediated PBF deletion (Fig. 3E and F; Supplementary Fig. 1). Src overexpression significantly repressed RAIU in parental MCF-7 cells transiently transfected with NIS (30%, *P* < 0.001) (Fig. 3G and H). In contrast, two PBF-knockout (PBF-KO) cell lines generated using different guide RNAs did not respond to Src overexpression (Fig. 3G and H). Likewise, Src overexpression resulted in a significant reduction in RAIU in parental MDA-MB-231 cells (36%, *P* < 0.001) but not in cells lacking PBF expression (Fig. 3I and J). RAIU was similarly repressed by Src in parental TPC-1 thyroid cancer cells but not CRISPR-Cas9 PBF-KO TPC-1 cells (19%, *P* < 0.001) (Supplementary Figs 1 and 4). Overall, these data demonstrate that Src overexpression represses NIS function in both breast and thyroid cancer cells in a manner entirely dependent upon PBF.

### Src myristoylation inhibition induces RAIU via PBF

**Figure 4:**
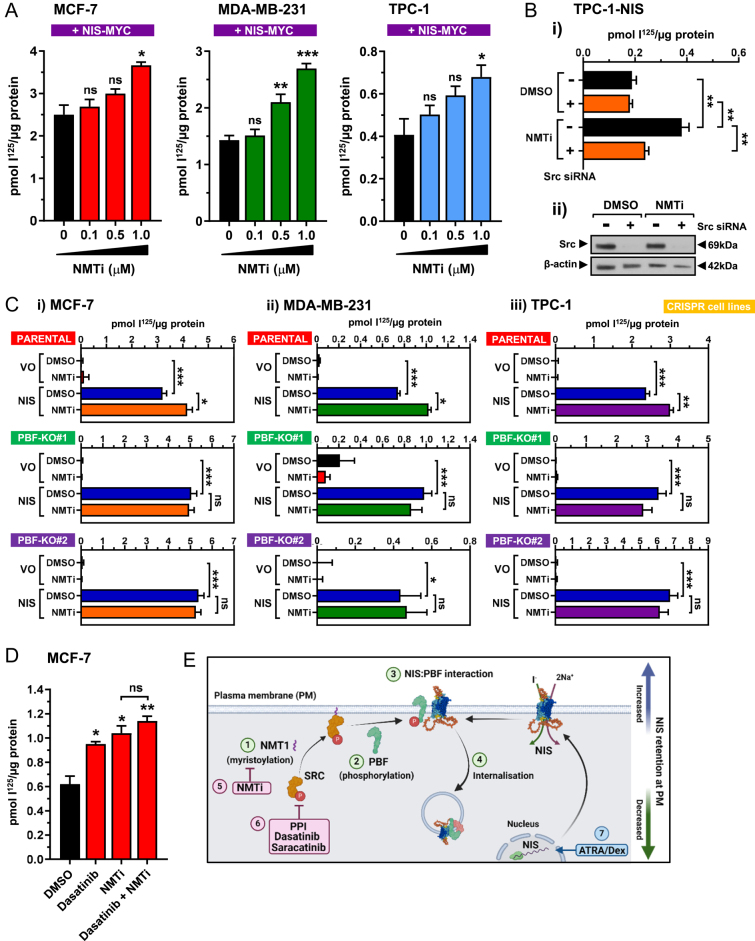
(A) RAIU in MCF-7 and MDA-MB-231 breast cancer cells and TPC-1 thyroid cancer cells transfected with NIS-MYC for 48 h and treated with varying doses of an NMT inhibitor (NMTi; 0–1 μM). The breast cancer cells were treated with NMTi for 4 h before RAIU and thyroid cancer cells were treated for 24 h. (B) RAIU in TPC-1 cells with stable NIS expression transfected with Src siRNA (or scrambled control) for 24 h before treatment with 1 μM NMTi (or DMSO vehicle control) for a further 24 h. (C) RAIU in parental MCF-7 (i), MDA-MB-231 (ii) and TPC-1 (iii) cells and two corresponding PBF knockout cell lines (PBF-KO #1 and #2) for each following VO or NIS transfection (48 h) and DMSO or 1 μM NMTi treatment (4 h – MCF-7/MDA-MB-231; 24 h – TPC-1). (D) TPC-1 cells were transfected with NIS-MYC for 48 h and then treated with DMSO, 1 nM dasatinib, 1 μM NMTi or a combination of the two drugs for 24 h before RAIU. (E) Schematic showing the NMT1/Src/PBF/NIS pathway. NMT1 myristoylates Src (1) and facilitates Src PM localisation and activation, resulting in PBF phosphorylation at Y174 (2). Phospho-PBF binds to NIS at the PM (3) leading to NIS internalisation and reduced RAIU (4). NMT1 inhibition (5) and Src inhibition (6) prevent PBF phosphorylation and NIS repression, while ATRA/Dex induces NIS expression and RAIU (7). *n* = 3 for all experiments. Error bars = SEM. Significance shown compared with VO and/or DMSO control unless otherwise shown. ns = not significant (*P* > 0.05), * = *P* < 0.05, ** = *P* < 0.01, *** = *P* < 0.001.

Src localisation and activity are mediated by N-myristoylation, the irreversible post-translational addition of a 14-carbon saturated fatty acid, myristate, to Src glycine residue 2 (31). Src myristoylation is catalysed by the N-myristoyltransferase NMT1 (32). To determine whether RAIU could be modified through targeting Src myristoylation, we employed an NMT inhibitor (DDD85646; NMTi). Both breast and thyroid cancer cells were treated with varying NMTi doses (0–1 μM). All cell lines showed a significant dose-dependent increase in RAIU, with maximal induction at 1 μM NMTi (MCF-7 ↑47%, *P* = 0.003; MDA-MB-231 ↑88%, *P* < 0.001; TPC-1 ↑67%, *P* = 0.036) (Fig. 4A).

We next assessed whether NMTi induced RAIU specifically through Src myristoylation inhibition. In TPC-1 cells with stable NIS expression, NMTi induced RAIU by 99% (scrambled control (Scr) + NMTi vs Scr + DMSO, *P* = 0.002). However, following siRNA-mediated Src knockdown, NMTi treatment was unable to stimulate RAIU (Src siRNA + NMTi vs Scr + DMSO, *P* = 0.308) (Fig. 4B).

To determine whether NMTi induction of RAIU was dependent upon PBF, we utilised the PBF-KO cell lines. Basal RAIU in parental MCF-7 cells was not stimulated by NMTi (Fig. 4C(i)) or dasatinib treatment (Supplementary Fig. 5A), further suggesting that these drugs influence NIS post-translationally. However, NMTi treatment resulted in a 29% increase in RAIU via exogenous NIS (*P* = 0.018) (Fig. 4C(i)). In contrast, neither of the two MCF-7 PBF-KO cell lines responded to NMTi. Similarly, PBF was required for NMTi induction of MDA-MB-231 and TPC-1 cells as evidenced by the loss of response in all PBF-KO lines tested (Fig. 4C(ii–iii)).

Given that Src therefore mediates NMTi induction of RAIU, we looked to further boost this effect through combination treatment of 1 μM NMTi and 1 nM dasatinib. However, dasatinib treatment did not further induce the NMTi-mediated increase in RAIU via exogenous NIS in any of the cell lines (Fig. 4D and Supplementary Fig. 5B and C). Similarly, although NMTi significantly induced ATRA/Dex-stimulated RAIU in MCF-7 cells, dasatinib treatment did not have an additive effect (Supplementary Fig. 5D), indicating that these inhibitors are targeting different parts of the same pathway (Fig. 4E).

Overall, these data show that NMT inhibition significantly induces RAIU in both breast and thyroid cancer cell lines. Src myristoylation inhibition mediates this effect, which is dependent upon PBF. These studies therefore identify a new class of drugs that induce RAIU *in vitro* through acting on NMT1 to inhibit Src-mediated PBF phosphorylation (Fig. 4E).

### NMT1 and Src expression are associated with recurrence in RAI-treated thyroid cancer

Given the availability of clinical data regarding radioiodide treatment, we first investigated NMT1, Src and PBF expression in thyroid cancer using The Cancer Genome Atlas (TCGA) and GEO datasets. NMT1, PBF and Src were all significantly upregulated in the TCGA PTC (THCA) cohort (Supplementary Fig. 6A) and in an independent GEO PTC dataset GSE60542 (Supplementary Fig. 6B). PBF and Src were highly expressed in the more aggressive BRAF-like PTC in comparison with RAS-like PTC and demonstrated a significant positive correlation in the entire PTC cohort (Supplementary Fig. 6C and D). In addition, increasing levels of NMT1 and Src expression were seen in PDTC and ATC, which had concomitantly lower levels of NIS expression (Supplementary Fig. 6E) (28). Of particular significance, NMT1 expression, in contrast to Src, was elevated in recurrent BRAF-like PTC treated with RAI, with ROC analysis indicating that high tumoural NMT1 might be a good predictor of recurrence (AUC = 0.734; Supplementary Fig. 6F).

Indeed, higher NMT1 and Src expression were associated with a significant reduction in DFS in RAI-treated PTC but not in non-RAI-treated PTC (Supplementary Fig. 7A, B, C). Cox regression analysis revealed that high tumoural NMT1 and Src were associated with an increased risk of recurrence in most subgroups except non-RAI-treated PTC (Supplementary Fig. 7D and E). Importantly, only RAI-treated PTC patients with high tumoural NMT1 and Src expression had significantly lower DFS compared with those with low NMT1 and Src expression (Supplementary Fig. 7F, G, H). RAI-treated PTC patients with low NIS expression in addition to high NMT1 or Src expression had the shortest DFS. However, DFS in patients with low NIS and high NMT1/Src was ∼40% lower than those with low NMT1/Src regardless of NIS expression (Supplementary Fig. 7I, J, K).

Overall, bioinformatic TCGA analyses demonstrated a strong association between high NMT1 and Src expression, increased recurrence risk and decreased DFS in PTC. The strength of these associations in RAI-treated patients may suggest a contribution to poorer therapeutic response.

### Association between high PBF and Src expression and more aggressive breast cancers

**Figure 5:**
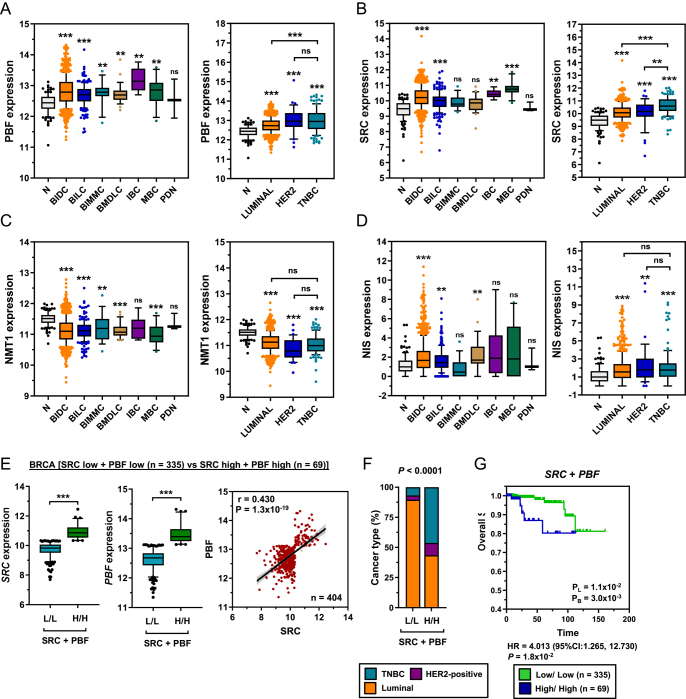
Elevated PBF and SRC expression in breast cancer. Box and whisker plots showing expression (log_2_) of PBF (A), SRC (B), NMT1 (C) and NIS (D) in various histotypes and molecular subtypes of the TGCA breast cancer (BRCA) RNA-Seq dataset versus normal (*N*; *n* = 112). BIDC = breast invasive ductal carcinoma (*n* = 812); BILC = breast invasive lobular carcinoma (*n* = 206); BIMMC = breast invasive mixed mucinous carcinoma (*n* = 16); BMDLC = breast mixed ductal and lobular carcinoma (*n* = 28); IBC = invasive breast carcinoma (*n* = 6); MBC = metaplastic breast cancer (*n* = 14); PDN = Paget’s disease of the nipple (*n* = 3); Luminal (ER^+^ and/or PR^+^; *n* = 565); HER2 = HER2-positive (*n* = 37); TNBC = triple negative breast cancer (*n* = 114). (E) *Left* – Box and whisker plots showing SRC and PBF expression in the BRCA cohort stratified by high versus low tumoural PBF/SRC expression in combination. *Right* – Correlation between those with high versus low tumoural PBF/SRC expression (*n* = 404/660). (F) *Left* – Proportion of molecular subtypes (%) within tumours with high versus low expression of both SRC and PBF. *Right* – Kaplan–Meier analysis of overall survival for BRCA patients with high versus low tumoural SRC and PBF expression. Number (*n*) of patients per sub-group. *P*_L_ = log-rank test; *P*_B_ = Breslow test. ns = not significant; ** = *P* < 0.01; *** = *P* < 0.001.

We next appraised the TCGA breast cancer (BRCA) dataset. Both PBF and Src expression were significantly upregulated in most BRCA histotypes and in all forms stratified by hormone status (oestrogen receptor (ER), progesterone receptor (PR), and human epidermal growth factor receptor 2 (HER2); Fig. 5A and B). Importantly, PBF and Src were most highly expressed in the more aggressive HER2-positive (ER^−^/PR^−^/HER2^+^) and TNBC (ER^−^/PR^−^/HER2^−^) tumours, which were associated with a significantly lower DFS compared with the ER^+^ and/or PR^+^ luminal cancers (Supplementary Fig. 8A). In contrast to THCA, NMT1 expression in the BRCA cohort was lower compared with normal breast tissue (Fig. 5C), although overall NMT1 expression levels were similar to thyroidal expression (Fig. 5 and Supplementary Fig. 6). NIS expression was also significantly upregulated in several histotypes, predominantly in the breast invasive ductal carcinoma (BIDC) and within all molecular subtypes (Fig. 5D).

Critically, stratification of patient groups identified a strong correlation between those with high versus low tumoural PBF and Src expression in combination (Fig. 5E, 404/660 BRCA patients). Of particular significance, patients with high PBF/Src expression had a significantly higher proportion of HER2-positive and TNBC tumours than those with low PBF/Src expression (Fig. 5F), which was reflected in greater reduction in overall survival (Fig. 5G) compared to other patient groups (Supplementary Fig. 8B). These findings indicate that PBF and Src are both associated with poorer survival characteristics in BRCA patients and represent promising new drug targets in patients who typically have aggressive disease and would benefit most from targeted enhancement of RAI therapy.

## Discussion

A major barrier to RAI utilisation as a potential breast cancer therapy is the lack of understanding of how NIS is trafficked within breast cancer cells. While NIS PM localisation is fundamental for RAIU, its regulation remains to be fully elucidated. Broadly, TSH promotes thyroidal NIS membrane localisation while the BRAF^V600E^ oncogene impedes it (11). Mass spectrometry-based interaction studies have been instrumental in identifying NIS interactors, such as valosin-containing protein (VCP), ADP ribosylation factor 4 (ARF4) (20), Rac family small GTPase 1 (RAC1) and ezrin (17). These studies demonstrate that cellular processes such as endoplasmic reticulum-associated degradation (ERAD) (20), endocytosis (12), actin cytoskeleton regulation (17) and cell–cell adhesion (18) govern NIS trafficking and PM retention. Furthermore, examples of universal NIS trafficking mechanisms, e.g. ARF4 and VCP regulation, exist in both breast and thyroid cancer cells (20).

PBF was the first NIS-interacting protein identified as a modulator of NIS subcellular localisation (13). Through NIS internalisation, PBF is a potent repressor of RAIU in thyroid cancer cells (13, 16), and we now show that PBF can also bind and repress NIS in breast cancer cells. Although PBF-Y174 is known to mediate the NIS/PBF interaction, putative binding sites on NIS, such as the PDZ-binding motif that is required for NIS PM localisation, are yet to be established (16, 33). Furthermore, we identified the SFK inhibitors saracatinib and dasatinib as potent PBF-pY174 inhibitors capable of reversing PBF-mediated NIS repression in breast cancer cells. Notably, Src overexpression significantly repressed RAIU and enhanced PBF repression of NIS, highlighting the substantial contribution of Src in modulating NIS.

To explore an alternative method of inhibiting Src, we targeted Src N-myristoylation, a key post-translational modification. NMT1-mediated attachment of myristate to the N-terminal end of Src is required for its membrane association and NMT1 inhibition/knockdown decreases Src activity (32, 34, 35, 36). Here, we demonstrated a dose-dependent NMTi induction of RAIU via exogenous NIS in both breast and thyroid cancer cells. Importantly, as ∼3% proteins are myristoylated (37), we used siRNA-mediated knockdown to confirm that NMTi-induced RAIU is Src-dependent. In addition, combinatorial NMTi and dasatinib treatment did not have an additive effect, suggesting that both induce RAIU via Src inhibition within the same pathway. Finally, the dependency of NMTi-induced RAIU on PBF was demonstrated using CRISPR-Cas9 PBF-knockout cell lines. Overall, these studies strongly suggest that Src represses RAIU through post-translational NIS modulation in a manner dependent on Src myristoylation and kinase activity, and through PBF phosphorylation.

Recently, another group verified our association between Src and NIS PM localisation following the identification of Src as a potential NIS interactor through mass spectrometry (17, 18). However, in contrast to our studies, Faria *et al* proposed that Src induces NIS PM localisation through activating the small GTPase RAC1. RAC1 signalling via p21-activated kinase 1 (PAK1) and phosphatidylinositol-4-phosphate-5-kinase (PIP5K)-induced ARP2/3-mediated actin polymerisation and recruitment of another NIS interactor, ezrin (17). While a clear interaction between Src and NIS was demonstrated, Src induction of RAIU was determined by treatment with PP2, which is a non-selective SFK inhibitor (38), and may therefore inhibit RAC1 signalling via other targets. However, both opposing pathways may be operational in cancer cells and further mechanistic investigations are required. Importantly, we have now shown that Src overexpression inhibits RAIU in multiple cell lines, and that several Src inhibitors, with different mechanisms of action, induce RAIU. In demonstrating a role for the Src substrate PBF in Src-mediated repression of RAIU, and its subsequent pharmacological restoration, these studies indicate a negative effect of Src on RAIU.

In order to relate these findings to clinical outcomes, we first performed a comprehensive analysis of TCGA and GEO thyroid cancer gene expression datasets, alongside PDTC and ATC datasets (28). We observed a strong association between high NMT1 and Src expression, more aggressive tumours, increased recurrence risk and decreased DFS in PTC. An association between increased Src expression/activity and thyroid tumour aggressiveness is well-established, with Src inhibition reducing thyroid cancer cell proliferation and invasiveness *in vitro* and tumour growth and metastasis *in vivo* (39, 40, 41, 42, 43, 44). While combined Src and MAPK pathway inhibitors are effective in both *in vitro* and pre-clinical studies (45, 46, 47), kinase inhibitor resistance is likely to limit clinical utility. Src inhibitor resistance is mediated by both MAPK and PI3K signalling pathway activation (46, 47, 48). However, combination therapy of Src, MAPK and PI3K inhibitors required to overcome resistance is likely to be too toxic in a clinical setting (49).

Increased NMT1 expression and activity is observed in multiple cancer types (31). Association with poor outcomes is considered to be due to aberrant Src activation, leading to increased cancer cell metabolism, cancer metastasis and drug resistance, presenting NMT1 as a potential therapeutic target (31). To our knowledge, this is the first report of NMT1 upregulation in thyroid cancer and we propose that high NMT1 expression predicts recurrence. High levels of both NMT1 and Src expression were associated with decreased DFS in RAI-treated patients, implying contribution to thyroid tumour progression. Analysis of the TCGA breast cancer cohort showed that NMT1 mRNA expression was reduced compared with normal tissue, although expression levels remained comparable with thyroidal NMT1. In contrast, a recent study showed that NMT1 is highly expressed in breast cancer and associated with aggressiveness (50). Furthermore, treatment with a pan-NMT inhibitor, PCLX-001, decreased breast cancer cell viability *in vitro* and led to a dose-dependent reduction in the growth of MDA-MB-231 breast cancer xenografts, demonstrating significant NMT1 activity and supporting potential clinical utility in breast cancer (50).

In the TCGA breast cancer cohort, there was a broad upregulation of PBF and Src across most tumour types. PBF is upregulated in breast tumours and a strong correlation exists between the number of repeat oestrogen-responsive elements (EREs) in the PBF promoter, PBF expression and breast cancer risk (19, 51). PBF also induces breast cancer cell migration and invasion (19, 52). Oncogenic Src overexpression and/or activation in breast cancer co-ordinates signal transduction between multiple membrane proteins and downstream targets to drive cell growth, survival, migration, invasion and, ultimately, tumour progression (53). Importantly, this study highlighted an association between high PBF and Src expression, HER2-positive and TNBC tumours, and decreased survival. Thus, in the most aggressive, difficult-to-treat breast tumours, which require alternative therapeutic options, targeting Src and PBF will likely be critical for efficacious RAI therapy.

As shown in multiple other studies (54), NIS expression was significantly upregulated in the TCGA breast cancers. Given the heterogeneous level of expression, it is likely that combinatorial treatment inducing both NIS expression and PM localisation will be most efficacious in breast cancer. Short-term enhancement of endogenous NIS expression in breast cancer cells using ATRA/Dex (29, 55) or HDAC inhibitors (56, 57, 58) with induction of PM NIS through NMT1/Src inhibition to cooperatively promote NIS function before giving RAI represents a promising treatment option that would overcome potential toxicity issues with long-term administration of these and other drugs. Of note, the inherent ability of the thyroid to avidly accumulate iodide would both hinder effective RAI targeting to breast tumours and potentially result in thyroidal damage, leading to hypothyroidism and increased risk of thyroid cancer. However, thyroidal uptake and accumulation of RAI can be selectively blocked using liothyronine (T3) to suppress TSH, resulting in reduced thyroidal NIS expression and function, and methimazole (MMI) to inhibit thyroid peroxidase (TPO) and prevent iodide organification (7). Therefore, pre-treatment with T3/MMI before RAI therapy would both avoid potential thyroid damage and maximise RAI availability to target breast tumours (7).

Overall, these studies have shown that PBF repression of NIS function can be modulated by Src and that RAIU may be enhanced through inhibiting Src myristoylation by NMT1. High expression of NMT1 and Src are associated with thyroid tumour recurrence and PBF and Src are highly expressed in breast cancer, particularly in more aggressive forms. Therefore, in advanced tumours that may benefit from alternative therapies, targeting NIS repression by Src and PBF through NMT1 inhibition may be a viable approach for augmenting RAI-avidity in breast cancer.

## Supplementary materials



## Declaration of interest

The authors declare that there is no conflict of interest that could be perceived as prejudicing the impartiality of the work reported.

## Funding

This work was supported by the Medical Research Council (MRC Doctoral Training Grant), the Government of Saudi Arabia (Scholarship NJU161) and the Department of Defense (grant number BC201532P1).
