# Supplementary material for: Targeting Src tyrosine kinase to enhance radioiodide uptake in breast cancer
Source: Endocr Relat Cancer. 2025 Aug 20;32(8):e240312. doi: 10.1530/ERC-24-0312 (PMC12372067; doi:10.1530/ERC-24-0312)
Supplement: Supplementary file 1 [file supplementary_materials.pdf]

Supplementary Figure 1

A

	gRNA including PAM	F Oligo	R Oligo
1	TCCAGTACGGCGTCGGCCCG**CGG**	CACCGTCCAGTACGGCGTCGGCCCG	AAACCGGGCCGACGCCGTACTGGAC
2	CCGTACTGGAGGTTGCGCCT**CGG**	CACCGCCGTACTGGAGGTTGCGCCT	AAACAGGCGCAACCTCCAGTACGGC

B

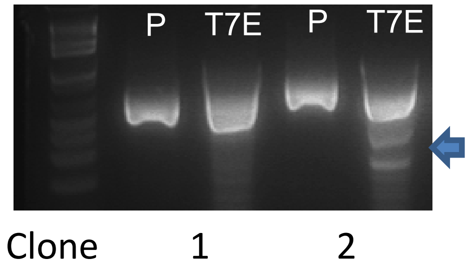

C

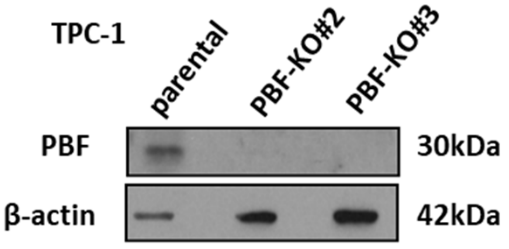

Figure S1. CRISPR-Cas9-mediated deletion of PBF. **A.** Two single guide RNA sequences (sgRNA) were designed to target PBF exon 1. For each gRNA the F and R oligos were annealed and cloned into the pLentiCRISPR vector before transfection into the parental cell lines. **B.** Example of the T7E mismatch assay used to screen cell clones. The target region was PCR amplified and the PCR product denatured and reannealed with wild-type DNA to induce heteroduplexes where mutations occurred. The T7 endonuclease (T7E) cleaves heteroduplexes and, when run on an agarose gel alongside the untreated PCR product (P), the cleavage products identify mutations (arrowed). **C.** Confirmation of PBF deletion by Western blotting in TPC-1 PBF-KO cells generated using gRNA#1 (PBF-KO #1) or gRNA#2 (PBF-KO #2).

Supplementary Figure 2

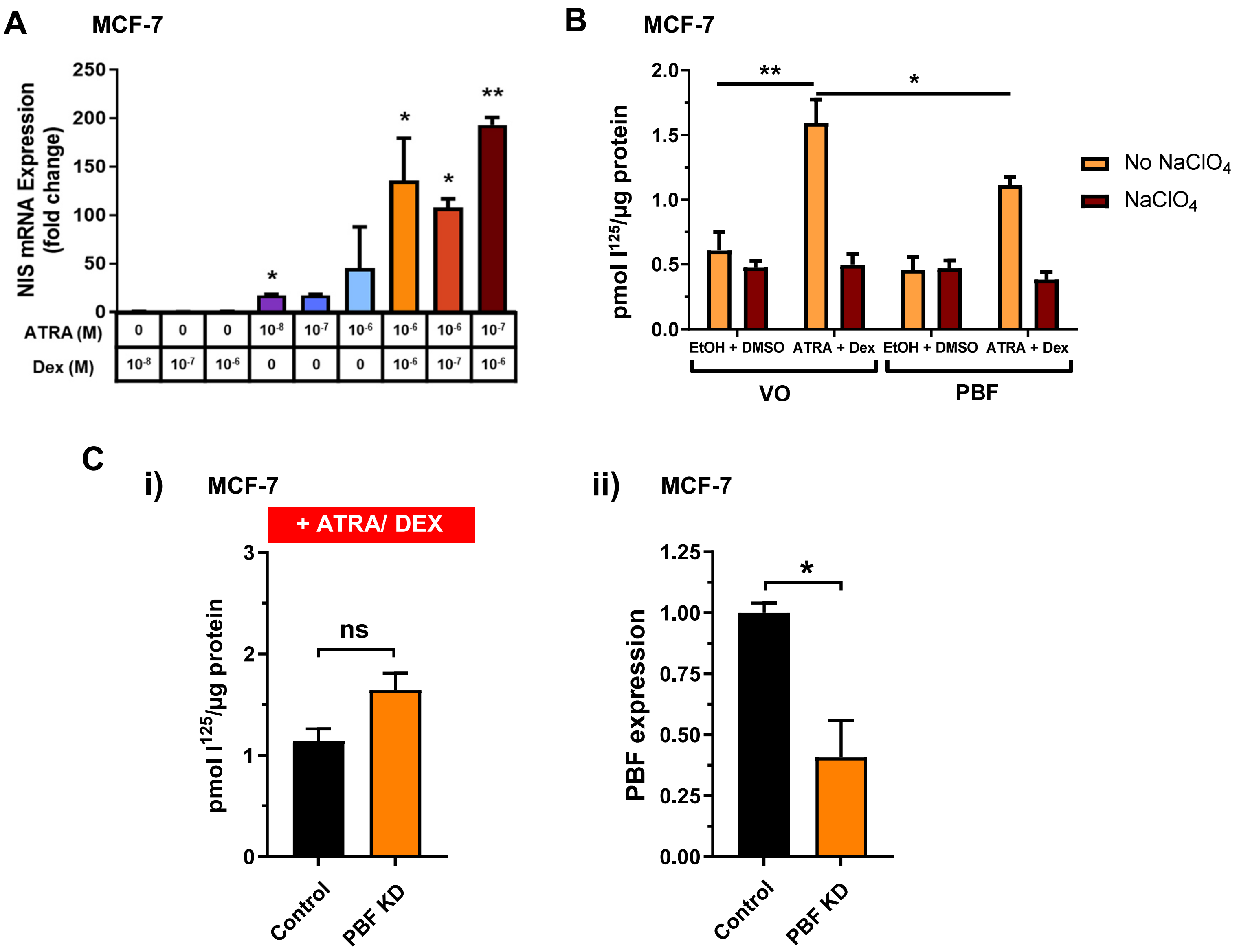

Figure S2. A. Treatment with ATRA/Dex increases NIS mRNA expression in MCF-7 cells. MCF-7 cells were treated with varying doses of ATRA and Dex for 48 hours and analysed by qPCR for NIS mRNA expression using 18s as the internal housekeeping gene. Combined 100 nM ATRA and 1 µM Dex treatment was determined as optimal and used in all subsequent experiments. Graph displays fold change compared with vehicle-only (ethanol and DMSO) treated cells. Significance shown compared with vehicle-only control and performed on ΔCT values. **B.** PBF decreases RAIU in ATRA/Dex-treated MCF-7 cells. MCF-7 cells stably transfected with either control empty vector (VO) or PBF were treated with ethanol and DMSO (vehicle) or 100 nM ATRA and 1 µM Dex for 48 hours prior to the addition of RAI. Sodium perchlorate ($NaClO_4$) controls demonstrate NIS-specific activity. **C. (i)** MCF-7 cells lentivirally-transduced with control or PBF shRNA were treated with ATRA/Dex for 48 hours before RAIU. **(ii)** Confirmation of PBF knockdown (KD) by qPCR. N=3. Error Bars = SEM. ns = not significant. * = $p<0.05$. ** = $p<0.01$.

Supplementary Figure 3

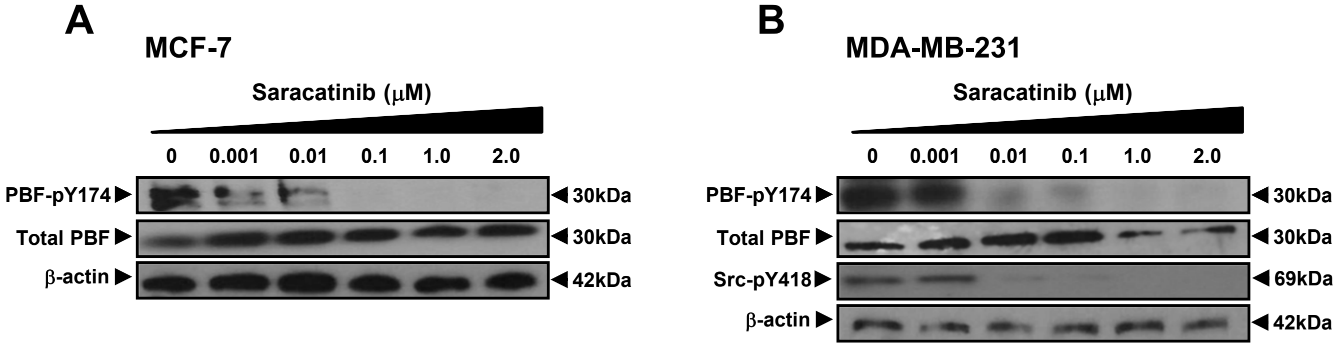

Figure S3. MCF-7 (**A**) and MDA-MB-231 (**B**) cells were treated with increasing doses of saracatinib (0-2 μM) for 24 hours before PBF-pY174, total PBF and Src-pY418 expression levels were determined by Western blot.

Supplementary Figure 4

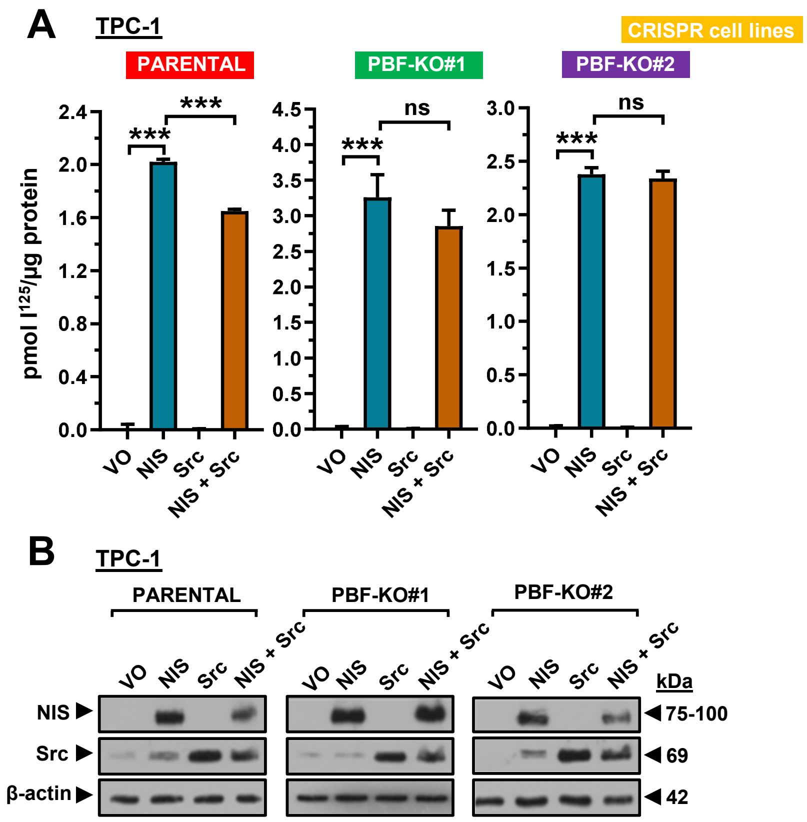

Figure S4. A. RAI uptake in parental TPC-1 cells and two PBF CRISPR knockout cell lines (PBF-KO #1 and #2) following NIS, Src and NIS + Src overexpression. **B.** Confirmation of successful transfection shown below by Western blotting. N = 3 for all experiments. Error Bars = SEM. Significance shown compared with VO unless otherwise shown. ns = not significant ($p > 0.05$), *** = $p < 0.001$.

Supplementary Figure 5

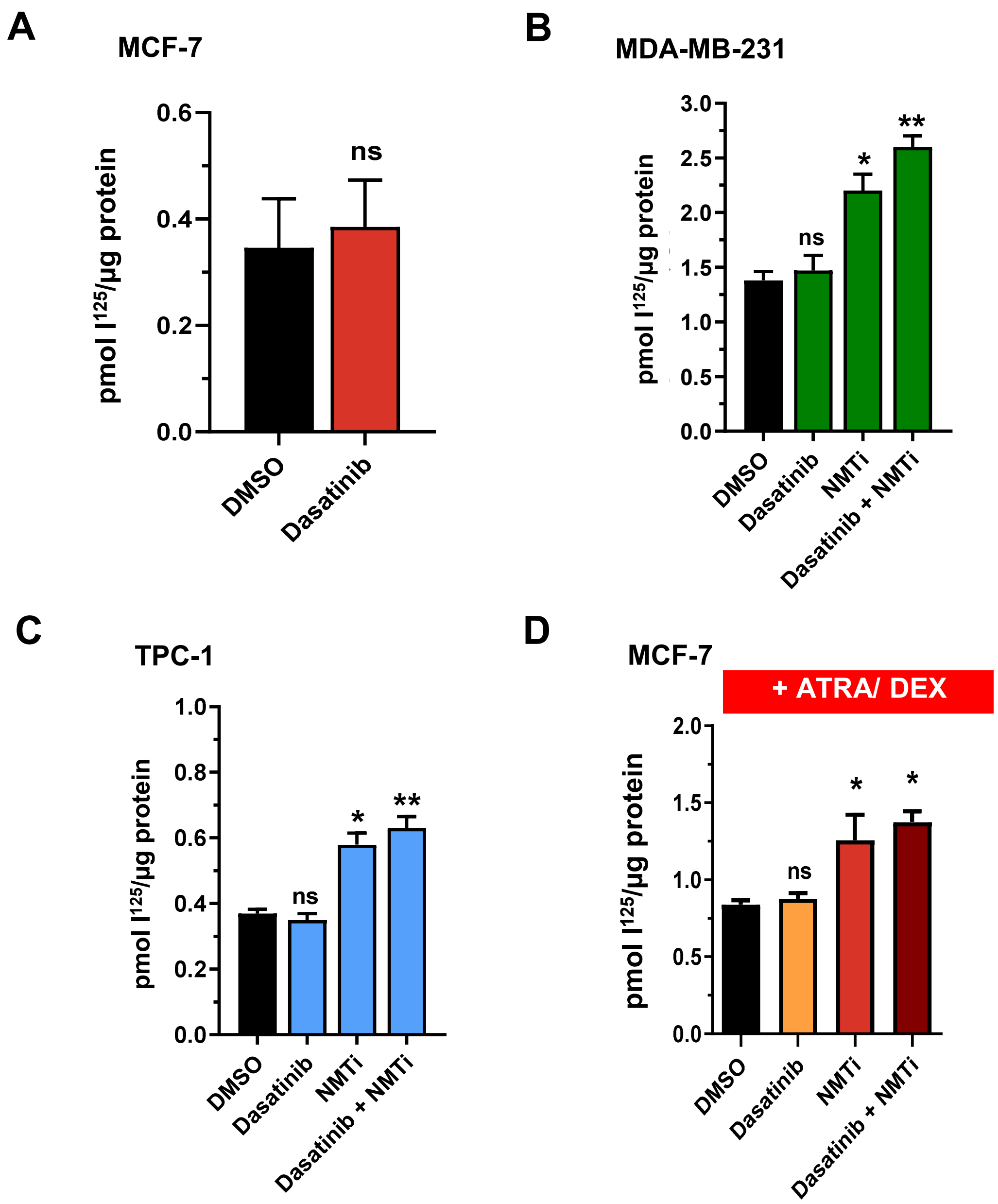

Figure S5. A. Basal RAIU in parental MCF-7 cells treated with DMSO vehicle control or 1 nM dasatinib for 24 hours. MDA-MB-231 (**B**) and TPC-1 (**C**) cells were transfected with NIS-MYC for 48 hours and then treated with DMSO, 1 nM dasatinib, 1 µM NMTi or a combination of the two drugs for 24 hours (dasatinib and NMTi in thyroid cells) or 4 hours (NMTi in breast cells) prior to RAI uptake. **D.** Similarly, ATRA/Dex stimulated-RAIU in MCF-7 cells was determined following dasatinib and/or NMTi treatment. N = 3-4. Error Bars = SEM. Significance shown compared with DMSO control. ns = not significant, * = $p < 0.05$, ** = $p < 0.01$.

Supplementary Figure 6

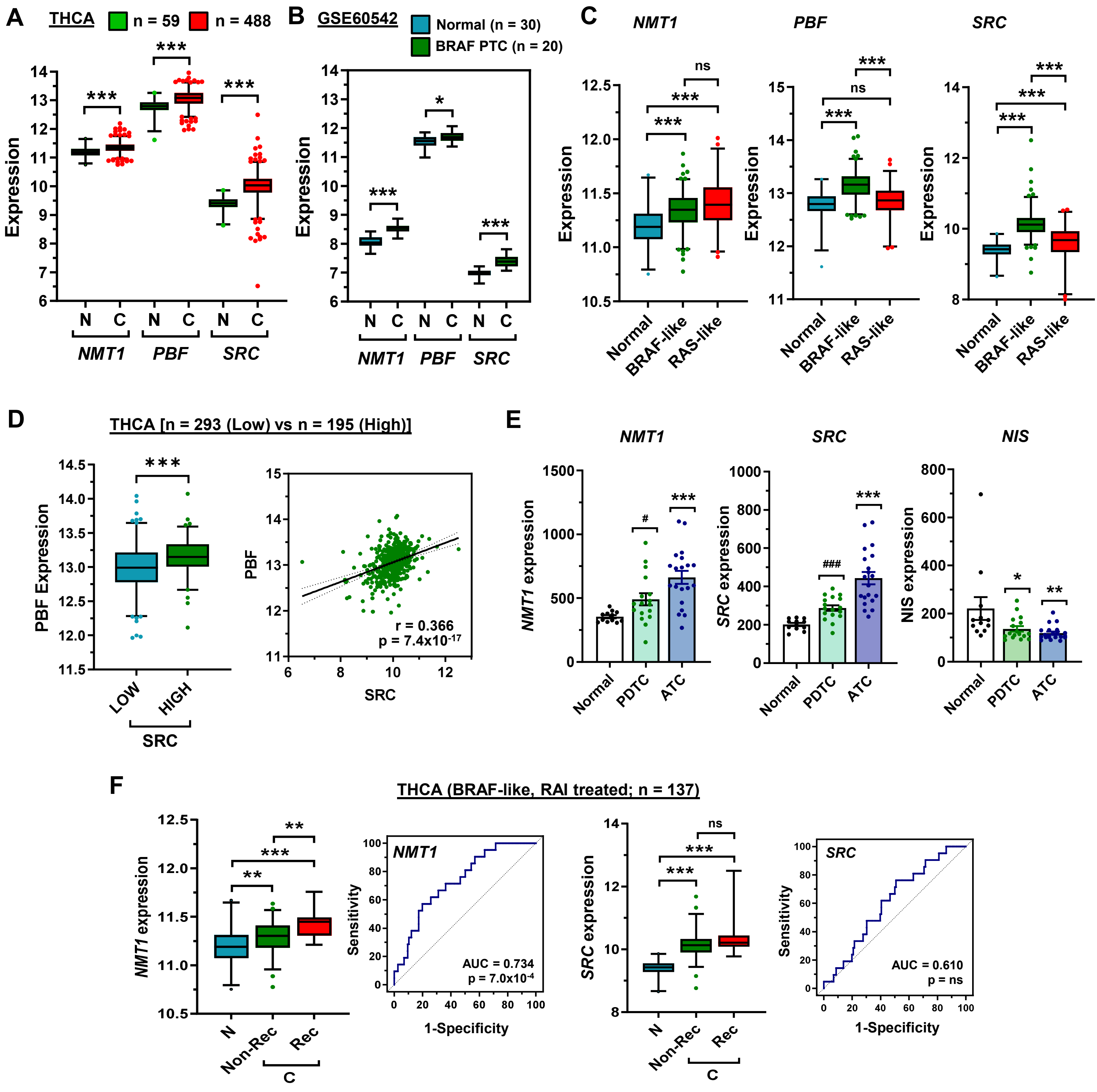

Figure S6. A and B. Box and whisker plots showing expression (log2) of NMT1, PBF and SRC genes in the **(A)** THCA (PTC) and **(B)** GSE60542 (BRAF PTC) cancer datasets (C) versus normal (N). **C.** Same as **(A)** but in BRAF-like (n=261) and RAS-like (n=116) THCA cohorts versus normal (n=59). **D.** PBF expression (log_2) in the THCA cohort stratified by high versus low SRC tumoural expression. *Right* - correlation between PBF and SRC expression in the THCA cohort (n=488). **E.** NMT1, SRC and NIS expression in normal thyroid (n=12), PDTC (n=17) and ATC (n=20). **F.** Box and whisker plots showing NMT1 and SRC expression in the BRAF-like, RAI-treated THCA cohort [recurrent (REC; n=21) versus non-recurrent (NON-REC; n=116)] with associated ROC curves (BRAF-like, RAI-treated THCA cohort (n=137)). Number (n) of patients per sub-group and adjusted p-values are shown. ns = not significant; * = $p < 0.05$; ** = $p < 0.01$; *** = $p < 0.001$. Unpaired two-tailed t-test (# = $p < 0.05$; ### = $p < 0.001$).

Supplementary Figure 7

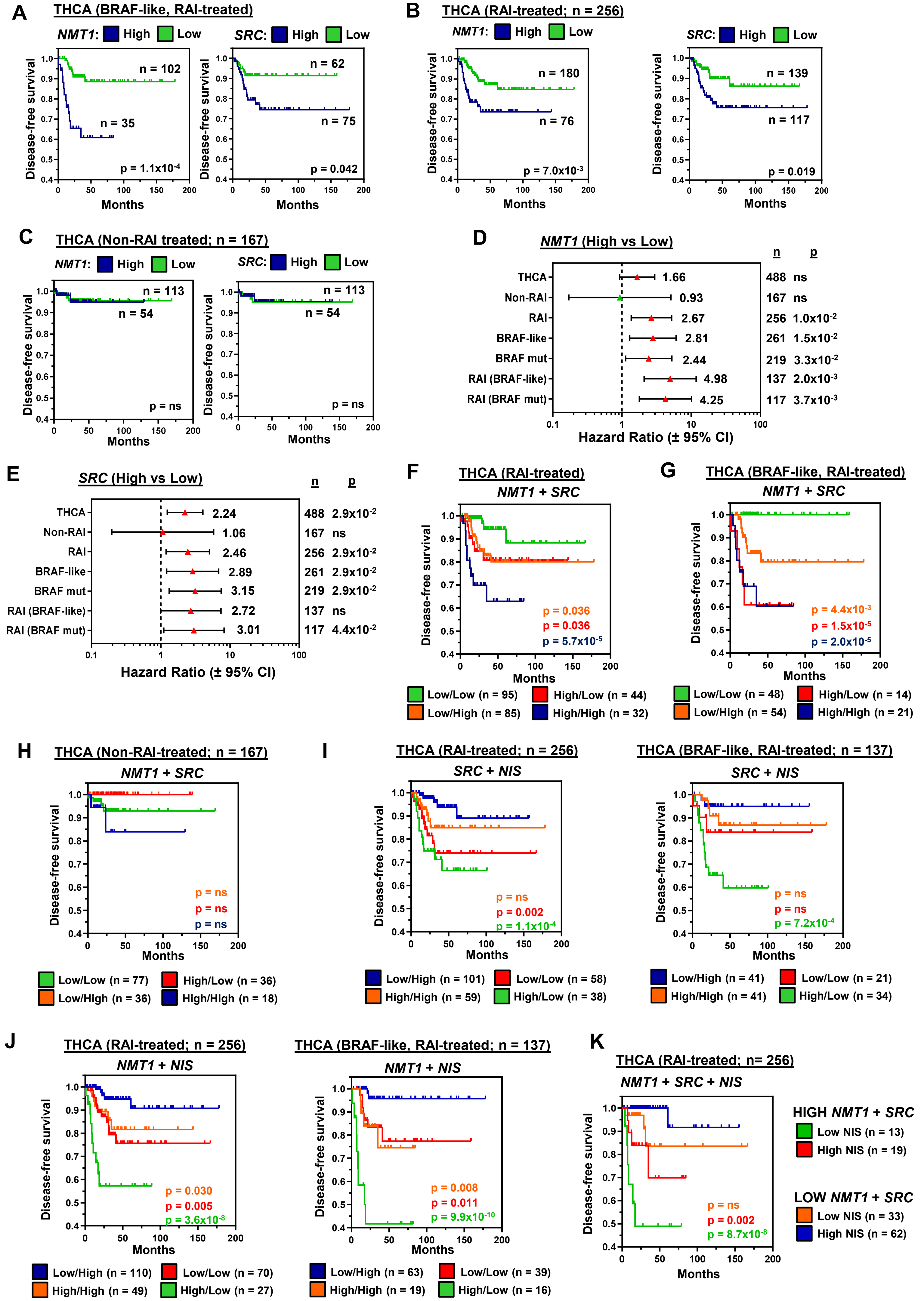

Figure S7. A-C. Kaplan-Meier analysis of DFS for NMT1 and SRC expression stratified by high versus low tumoural expression in the BRAF-like, RAI-treated THCA (**A**), RAI-treated THCA (**B**) and non-RAI treated THCA (**C**) cohorts; log-rank test. Number (n) of patients per sub-group and adjusted p-values are shown. **H** and **I**. Hazard ratio ± 95% CI for patients stratified by NMT1 (**H**) and SRC (**I**) expression in THCA with the indicated treatment and genetic signature or alteration. **F-L.** Representative Kaplan-Meier analyses of DFS for the indicated THCA cohorts stratified by high versus low tumoural expression of NMT + SRC (**F-H**), SRC + NIS (**I**), NMT1 + NIS (**J**) and NMT1 + SRC + SLC5A5 (NIS) (**K**); log-rank test. Number (n) of patients per sub-group and adjusted p-values are shown. ns = not significant; * = $p < 0.05$; ** = $p < 0.01$; *** = $p < 0.001$.

Supplementary Figure 8

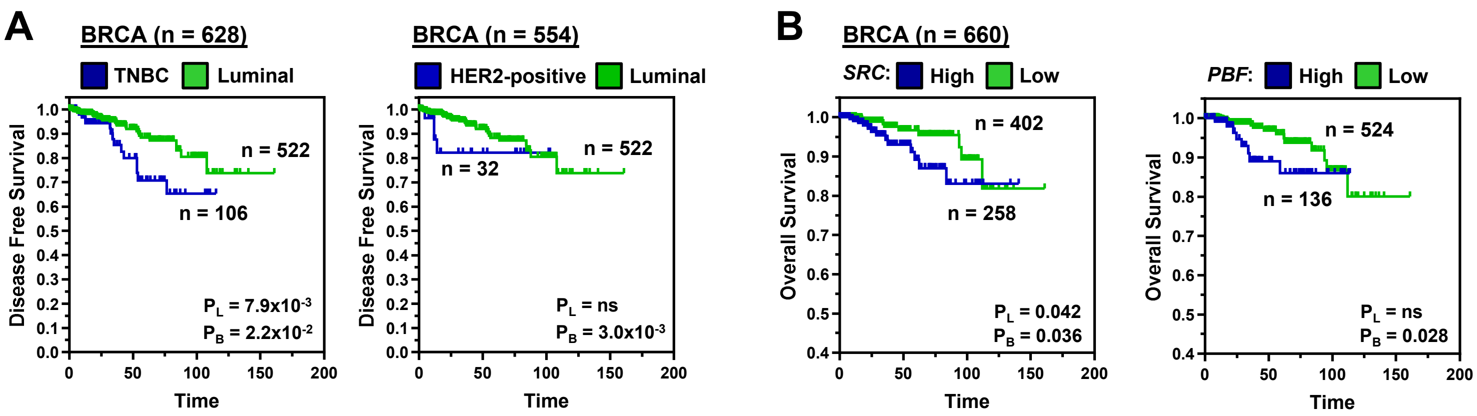

Figure S8. A. TCGA BRCA analysis. Kaplan-Meier analysis of DFS for the TNBC and HER2-positive versus luminal subtypes. **B.** Kaplan-Meier analyses of overall survival for the BRCA cohort stratified by high versus low tumoural expression of SRC and PBF. Number (n) of patients per sub-group; P_L = log-rank test; P_B = Breslow test. ns = not significant.
